# Manual acupuncture benignly regulates blood-brain barrier disruption and reduces lipopolysaccharide loading and systemic inflammation, possibly by adjusting the gut microbiota

**DOI:** 10.3389/fnagi.2022.1018371

**Published:** 2022-10-13

**Authors:** Yue Zhang, Ning Ding, Xin Hao, Jun Zhao, Yali Zhao, Yiran Li, Zhigang Li

**Affiliations:** ^1^School of Acupuncture, Moxibustion and Tuina, Beijing University of Chinese Medicine, Beijing, China; ^2^Department of Acupuncture, Guang’anmen Hospital, China Academy of Chinese Medical Sciences, Beijing, China; ^3^School of International, Beijing University of Chinese Medicine, Beijing, China

**Keywords:** Alzheimer’s disease, gut microbiota, lipopolysaccharide, blood-brain barrier, acupuncture

## Abstract

**Background:**

Blood-brain barrier (BBB) disruption and gut microbiota dysbiosis play crucial roles in Alzheimer’s disease (AD). Lipopolysaccharide (LPS) stimulation triggered by gut microbial dysbiosis is an important factor in BBB disruption and systemic inflammation, but the mechanism of acupuncture regulation of BBB disruption *via* the gut microbiota in AD is not clear.

**Objective:**

The current study evaluated the effect of manual acupuncture (MA) on BBB dysfunction in APP/PS1 mice and examined the mechanism of gut microbiota by acupuncture in AD.

**Methods:**

Acupoints were applied to Baihui (GV20), Yintang (GV29), and Zusanli (ST36) in the MA group. Mice in the manual acupuncture plus antibiotics (MAa) group received antibiotics and acupuncture, while mice in the probiotics (P) group received probiotics. Alterations in spatial learning and memory, the gut microbiota, tightly connected structure and permeability of BBB, and the expression of LPS and inflammatory factors in each group were assessed.

**Results:**

Compared to the normal (N) group, cognitive ability was significantly impaired, the gut microbiota composition was markedly altered, the BBB was significantly disrupted, and the expression of LPS in serum and brain, serum TNF-α, and IL-1β were significantly increased in the AD group (*p* < 0.01). These changes were inhibited in the MA and P groups (*p* < 0.01 or *p* < 0.05), and antibiotics reversed the benign regulatory effects of MA (*p* < 0.01 or *p* < 0.05).

**Conclusion:**

Manual acupuncture benignly modulated the gut microbiota and BBB dysfunction, reduced LPS, TNF-α, and IL-1β. These effects were comparable to probiotics. The decrease in LPS load and systemic inflammation may play important roles in the regulation of BBB dysfunction by acupuncture, and the gut microbiota may be a potential target for the benign regulation of BBB disruption by acupuncture.

## Introduction

Alzheimer’s disease (AD) is a form of dementia and a neurodegenerative disease that is characterized by progressive cognitive decline, specific neuronal apoptosis, and synaptic loss (Ozben and Ozben, 2019). There are currently more than 44 million AD patients worldwide, and this number is expected to triple by 2050 (Scheltens et al., 2021; Collaborators, 2022). The number of deaths from AD has increased 71% during this decade (Hodson, 2018). Because the aging of the population continues to accelerate, the economic burden imposed by AD is becoming heavier. This disease has become a worldwide public health problem that must be addressed (Marešová et al., 2015; Alzheimer’s Association Organization, 2021).

The main pathological features of AD are amyloid plaque deposition and neurofibrillary tangle formation (Mahaman et al., 2022), but an increasing number of studies found that these two pathologies are not the only causes of cognitive decline (Kobayashi et al., 2017; Pluta et al., 2020). BBB dysfunction occurs throughout AD onset and progression, and this alteration precedes the onset of AD pathology and clinical symptoms (Erickson and Banks, 2013). The BBB is composed of the vascular endothelium, basement membrane, and the surrounding peduncles of pericytes and astrocytes (Abbott et al., 2010; Yamazaki and Kanekiyo, 2017). Neuroinflammation, oxidative stress, and glucose transport disorder trigger alterations in endothelial function and tight junction structures. These changes directly or indirectly accelerate AD-related neurodegenerative changes (Aliev et al., 2011; Shah et al., 2012; Chen and Zhong, 2013). Recent studies found that BBB dysfunction in AD was closely related to intestinal flora dysbiosis (Braniste et al., 2014; Fung et al., 2017). Some gut microbial metabolites, such as short-chain fatty acids (SCFA) (MacFabe, 2015; Colombo et al., 2021), gamma-aminobutyric acid (GABA) (de J R De-Paula et al., 2018), and neurotrophic factors (GDNFs) (Caraci et al., 2012), are neurotransmitters that directly affected BBB integrity. The expression of these transmitters are contributes to the upregulation of brain endothelial tight junction proteins (Hoyles et al., 2018). Aging disrupts the balance of the intestinal microbiota, and harmful bacterial metabolites break through the intestinal epithelium and activate peripheral immune cells (De Maeyer and Chambers, 2021). BBB permeability ultimately decreases *via* the upregulation of adhesion molecules and matrix metalloproteinases (Parker et al., 2020). Peripheral macrophages and immune cells infiltrate into the brain, which aggravates neuroinflammation (Mark and Miller, 1999). Inflammation and BBB dysfunction inhibit the metabolic activity of the brain, which leads to the accumulation of neurotoxins (Bone, 2007). It is worth affirming that the BBB plays an important role as a mediator of communication between the gut microbiome and the brain.

A previous study found that LPS stimulation caused by gut microbial dysbiosis led to BBB dysfunction. The diversity and composition of the gut microbiota are altered in AD (Harach et al., 2017; Bäuerl et al., 2018). At the phylum level, Proteobacteria, Firmicutes, Bacteroidota, and Actinobacteriota were increased. At the genus level, Helicobacter and Escherichia were decreased (Cattaneo et al., 2017; Vogt et al., 2017; Wu et al., 2021). Gut microbes affect central nervous system (CNS) function *via* complex and diverse pathways, but the most important pathway, is the gut-brain axis (Kowalski and Mulak, 2019; Rutsch et al., 2020). LPS produced by Gram-negative bacteria is closely associated with AD-related neuroinflammation (Zhan et al., 2016; Zhao et al., 2017). LPS is a potent proinflammatory mediator that disrupts the intestinal epithelium, damages the intestinal mucosal barrier, and enters the periphery to activate the immune system and induce the release of proinflammatory factors, such as IL-1β and TNF-α, which cause systemic inflammation (Varatharaj and Galea, 2017; Haruwaka et al., 2019). This inflammatory state rapidly destabilizes endothelial structures (Huang et al., 2020; Wang et al., 2021b) and leads to the translocation of proteins associated with tightly connected structures, which greatly increases BBB permeability (Montagne et al., 2015; Liang et al., 2020; Nehra et al., 2022). These pathological alterations exacerbate the infiltration of peripheral inflammatory substances into the CNS and induce a series of cascade reactions that lead to progressive neurodegeneration and neuronal apoptosis (Zhao et al., 2015b; Janyou et al., 2017).

Our previous work demonstrated that acupuncture significantly improved spatial learning and memory in AD model animals (Tang et al., 2020; Xu et al., 2020), exerted anti-inflammatory effects (Jiang et al., 2018), modulated glucose metabolism (Cao et al., 2017), and improved cerebral blood flow by downregulating the astrocytic phospholipase A2 (PLA2)-arachidonic acid (AA) pathway (Ding et al., 2019a,b). Our team also confirmed that acupuncture upregulated the expression of microRNA-181a, which is closely related to the BBB (Wu et al., 2019), and cleared amyloid β-peptide (Aβ) *via* low-density lipoprotein receptor-related protein-1 (LRP1) in the BBB (Wang et al., 2016). These results suggest that the BBB is a key target of acupuncture in the treatment of AD. Previous studies have confirmed that the gut microbiota was an important target of acupuncture in the modulation of related diseases (Jang et al., 2020; Jiang et al., 2021). However, the effects of MA in modulating the BBB in APP/PS1 mice have not been clarified, and the gut microbiota-related mechanisms have not been elucidated. Therefore, changes in gut microbiota composition, LPS levels and BBB dysfunction in APP/PS1 mice were observed to elucidate the gut microbiota-related mechanism of MA regulation of the BBB. The current study provides a foundation for further in-depth investigation of the mechanism of AD and reveals the role of the gut microbiota in the alleviating effect of MA in AD.

## Materials and methods

### Experimental animals

Male APP/PS1 and male C57BL/6 mice were purchased from the Cavens Biogle Academy of Model Animal Research (Suzhou) (animal lot: SCXK-Su-2018-0002). The mice weighed 30.0 ± 2.0 g and were 6 months old. Water and food were provided *ad libitum*. All experimental procedures and animal welfare were approved by the Animal Ethics Committee of Beijing University of Chinese Medicine (ethics number: BUCM-4-2021102602-4031).

### Animal grouping and intervention

Eighty APP/PS1 mice were divided into four groups: the Alzheimer’s disease group (AD), the manual acupuncture group (MA), the manual acupuncture plus antibiotics group (MAa), and the probiotics group (P). Twenty C57BL/6 mice were used as the normal group (N). Based on our previous studies (Ding et al., 2019b), the acupoints included Baihui (GV20), Yintang (GV29), and Zusanli (ST36).

From days 1 to 7, mice in MAa group received antibiotics by oral gavage consisting of Clindamycin (150 mg/kg), Metronidazole (60 mg/kg), Vancomycin (25 mg/kg), Neomycin (60 mg/kg), and Ampicillin (50 mg/kg) (Hintze et al., 2014; Staley et al., 2017; Guida et al., 2018; Zeng et al., 2020). Then, from days 8 to 45, mice were given *ad libitum* access to water containing antibiotics (0.5 mg/ml Clindamycin, 1.0 mg/ml Metronidazole, 0.5 mg/ml Vancomycin, 0.5 mg/ml Neomycin, and 1.0 mg/ml Ampicillin) (Lamousé-Smith et al., 2011) and simultaneously treated with MA.

From days 8 to 45, in the MA group, disposable sterile acupuncture needles (Beijing Zhongyan Taihe Medicine Company, Ltd., China) were used in the acupoints for 20 min. GV20 and GV29 were adopted the transverse puncturing at a depth of 2–3 mm, ST36 was used the vertical puncturing at a depth of 4 mm. During the puncturing process, needle was twisted bidirectionally within 90° at a speed of 180°/s within 90°. Rotating manipulation was applied every 5 min for 15 s. The P group received probiotics (8.7 × 10^8^ CFU/g/day, containing *Bifidobacterium animalis* ssp. *lactis HN019, Bifidobacterium bifidum Bb06, Bifidobacterium animalis* ssp. *lactis BB-12, Bifidobacterium animalis* ssp. *lactis Bi07, Bifidobacterium longum R175, Bifidobacterium animalis B94, Lactobacillus rhamnosus GG, L. casei Lc11, Lactobacillus helveticus R52, Lactobacillus paracasei Lpe37, Lactobacillus plantarum R1012, Lactobacillus reuteri HA188, Lactobacillus rhamnosus R11, Lactobacillus acidophilus NCFM*, and *Streptococcus thermophiles St21*) (Beijing Zhongke Yikang Biotechnology Company Ltd., China) by oral gavage. The N, AD, and P groups received a restriction of 20 min.

### Animals handling and sample collection

On the first and seventh day of the experiment, eight mice in the MAa group were randomly selected for fresh feces collection. Fresh feces were collected from eight mice per group on day 46. Ten mice in each group were selected for the Morris water maze (MWM) test from days 40 to 45. Six mice in each group were assessed using Evans blue (EB) staining, and six mice per each group were used for enzyme-linked immunosorbent assays (ELISA) and Western blotting (WB). Six mice in each group were subjected to immunofluorescent (IF) staining, and two mice in each group were chosen for transmission electron microscope (TEM) assays. All animals were anesthetized using sodium pentobarbital (80 mg/kg).

### Morris water maze test

Mice were subjected to the hidden platform trials for 5 days. The platform was located in the middle of the southwest (SW) quadrant. Each mouse had 60 s to search for the underwater platform. If the platform was not found within 60 s, the mice were guided to swim up to the platform and stayed on it for 15 s. The escape latency and swimming speed were recorded using analysis system. The platform was removed after the hidden platform trials. Each mouse was subjected to the probe trial for 1 day. The mouse was placed in the northeast (NE) quadrant and the swimming path was recorded within 60 s. The percentage of time spent and distance swam in the target quadrant were analyzed.

### 16S rRNA

Fecal samples were collected and microbial DNA was extracted. The hypervariable region V3-V4 of the bacterial 16S rRNA was amplified by PCR using the 341F (5′CCTACGGGNGGCWGCAG-3′) and 805R (5′-GACTACHVGGGTATCTAATCC-3′). Purified amplicons were pooled in equimolar and paired-end sequenced on an Illumina MiSeq PE300 platform/NovaSeq PE250 platform (Illumina, San Diego, CA, USA) according to the standard protocols by Majorbio Bio-Pharm Technology Co., Ltd. (Shanghai, China) (Magoè and Salzberg, 2011; Edgar, 2013). The raw 16S rRNA gene sequencing reads were demultiplexed, quality-filtered by fastp version 0.20.0 and merged by FLASH version 1.2.7 (Chen et al., 2018). Operational taxonomic units (OTUs) with 97% similarity cutoff were clustered using UPARSE version 7.1, and chimeric sequences were identified and removed (Edgar, 2013).

### Transmission electron microscope assay

The cortical tissues (1 mm^3^) were fixed in 2.5% glutaraldehyde for 6 h. The tissue was rinsed in phosphate buffer and fixed in 1% osmium fixative for 2 h. After dehydration in an ethanol gradient, acetone embedding solution was added. Samples were placed in an oven for curing, sectioned at 50 nm on an ultrathin sectioning machine, and stained with 3% uranyl acetate-lead citrate. Ultrathin sections were obtained and stained with uranyl acetate (Ceafalan et al., 2019). Then, the tightly connected structure of the BBB was observed by TEM (FEI Tecnai Spirit, United States).

### Evans blue extravasation

After anesthetization, mice were perfused with 50 mL of 4% paraformaldehyde and fixed with 1% EB (Del Valle et al., 2008, 2009). The brains were dissected and dehydrated with 30% sucrose for 24 h. Frozen 10 μm sections were obtained using a freezing microtome (Leica, Germany) and observed at 40 × magnification under a laser confocal microscope (Olympus, Japan). The following regions of interest were analyzed: the CA1, CA2, CA3, and DG regions of the hippocampus and vascular endothelium. The mean optical intensity was analyzed by ImageJ.

### Immunofluorescent staining

The brains were dissected, fixed in 4% paraformaldehyde for 3 h, and dehydrated in 20 and 30% sucrose for 24 h each. Frozen 6 μm sections were sliced. The primary antibodies included rabbit polyclonal ZO-1 (1:100, Invitrogen, United States), rabbit polyclonal occludin (1:100, Invitrogen, United States), and mouse polyclonal LPS (1:100, Hycult Biotech, United States). Donkey anti-rabbit IgG Alexa Fluor 488 (1:200, Abcam, United States) and donkey anti-mouse IgG Alexa Fluor 594 (1:200, Abcam, United States) were used as the corresponding secondary antibodies. DAPI (Abcam, United States) was added to the sections for counterstaining. Samples were observed at 63 × magnification under laser confocal microscope (Leica, Germany). These sections were used to analyze the mean optical intensity of ZO-1, occludin, and LPS (Ahn et al., 2018; Liu et al., 2021).

### Western blotting

Hippocampal tissues were isolated from the brain and samples were centrifuged. After protein concentration was determined, samples were loaded into 8% SDS-PAGE gel wells for electrophoresis. Separated proteins were transferred to polyvinylidene difluoride (PVDF) membranes. After washing, bovine serum albumin (BSA) was added for 1 h at room temperature. Primary antibodies against ZO-1 (1:500, Invitrogen, United States), occludin (1:500, Invitrogen, United States), and β-actin (1:500, Bioss, United States) were added and incubated overnight at 4°C. Secondary antibodies conjugated to horseradish peroxidase (HRP) was added for 1 h at room temperature, and protein was detected using an ECL luminescent solution. The density of all WB bands was compared with that of the β-actin band, and β-actin was used as the internal control.

### Enzyme-linked immunosorbent assays

Blood samples were incubated at room temperature for 1 h and centrifuged, and the supernatant was collected. The antigen was diluted in an coating solution and the samples were incubated overnight at 4°C. The wells were washed, and a closure solution was added. The serum was diluted and added to the well plates. Horseradish peroxidase anti-mouse IgG was added for incubation for 2 h at room temperature. TMB reaction solution was added, and termination solution was mixed after reaction in a dark room. The concentration of LPS, TNF-α, and IL-1β were recorded.

### Statistical analysis

Statistical analysis was performed using SPSS26.0 (SPSS, Inc., Chicago, IL, United States). Data are expressed as the mean ± SD deviation. Hidden platform trial results and swimming speed results were analyzed by repeated-measures analysis. A one-way ANOVA followed by the LSD multiple-range test was used to compare normal distribution data and the homogeneity of variance. For the non-normally distributed data or data with heterogeneous variance, the Kruskal–Wallis test was used. The level of significance was set at *p* < 0.05.

## Results

### Effect of manual acupuncture on spatial learning and memory

**FIGURE 1:**
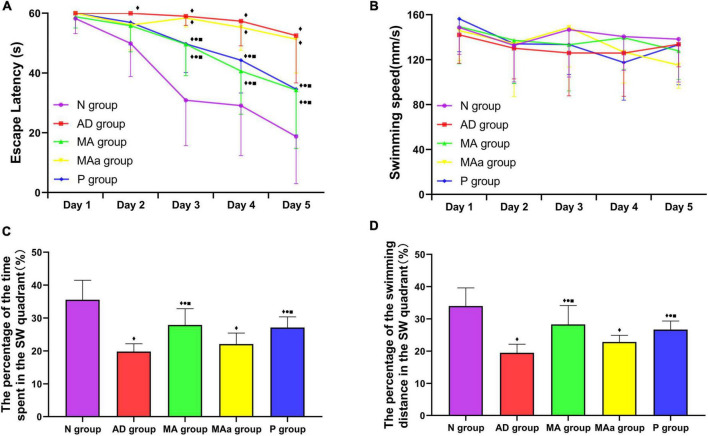
Results of the MWM test for each group (*n* = 10, mean ± SD). **(A,B)** Comparison of the escape latency and swimming speed of each group in the hidden platform trials. **(C,D)** Comparison between the percentage of time spent and swimming distances in the SW quadrant in each group. Compared to the N group, ^◆^*p* < 0.01 or *p* < 0.05. Compared to the AD group, ^•^*p* < 0.01 or *p* < 0.05. Compared to the MAa group, ^■^*p* < 0.01 or *p* < 0.05.

The results of the MWM test are presented in Figure 1. The escape latency of the N, MA, and P groups showed a decreasing trend from days 2 to 5, but there was no significant change in the escape latency of the AD or MAa group. The escape latency of the AD group was significantly higher than that of the N group from days 2 to 5 (*p* < 0.01). Compared to the AD group, the escape latency of the MA and P groups was significantly lower on days 3 to 5 (*p* < 0.01 or *p* < 0.05). Compared to the MAa group, the escape latency of the MA and P groups were decreased on days 3 to 5 (*p* < 0.01 or *p* < 0.05). No significant difference in swimming speed was observed between the groups.

The percentage of time spent and distance traveled in the target quadrant were significantly lower in the AD and MAa groups than the N group (*p* < 0.01). The percentage of time spent and distance traveled in the target quadrant in the MA and P groups were significantly higher than the AD and MAa groups (*p* < 0.01) but were lower than the Nr group (*p* < 0.01). There was no significant difference in these measures between the AD and MAa groups.

### Effect of manual acupuncture on the gut microbiota

**FIGURE 2:**
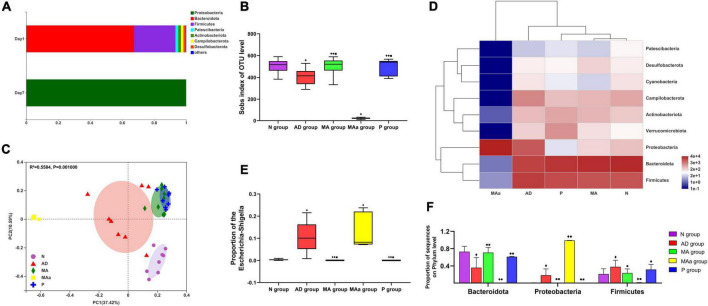
Results of 16S rRNA for each group (*n* = 8, mean ± SD). **(A)** Community barplot analysis in the MAa group. **(B,C)** Sobs index and PcoA analysis in each group. **(D)** Heatmap at the phylum level in each group. **(E,F)** Comparison of the abundance of phylum and genus in each group. Compared to the N group, ^◆^*p* < 0.01 or *p* < 0.05. Compared to the AD group, ^•^*p* < 0.01 or *p* < 0.05. Compared to the MAa group, ^■^*p* < 0.01 or *p* < 0.05.

Species diversity and composition data are shown in Figure 2. The phyla in the MAa group before antibiotic pretreatment primarily included Bacteroidota, Firmicutes, Patescibacteria, Actinobacteriota, Campyiobacterota, and Desulfobacterota, but only Proteobacteria was detected after antibiotic administration. The Sobs index was lower in the AD group and MAa groups than the N group (*p* < 0.01). Compared to the AD group and MAa groups, the Sobs index of the MA group and the Pr group was obviously increased (*p* < 0.01). Principal coordinate analysis (PCoA) indicated that the bacterial composition was distinct between the AD and MAa groups and the N, MA, and P groups. The bacterial composition was more similar between the MA group and the P group. The heatmap of abundance at the phylum level showed that the five phyla with high abundance were Bacteroidota, Firmicutes, Proteobacteria, Campilobacterota, and Actinobacteriota. The bacterial community was dominated by Bacteroidota, Firmicutes, and Proteobacteria in the AD group, with Firmicutes showing the highest abundance. Bacteroidota and Firmicutes were dominant in the MA, P, and N groups, with Bacteroidota exhibiting the highest abundance. The bacterial community was dominated by Proteobacteria in the MAa group. The abundances of Proteobacteria, Bacteroidota, and Firmicutes at the phylum level in each group were compared. The abundances of Proteobacteria and Firmicutes in the AD group were significantly higher than the N group (*p* < 0.01 or *p* < 0.05), and the abundance of Bacteroidota in the AD group was significantly lower than that in the N group (*p* < 0.01). Compared to the AD group, the abundances of Proteobacteria and Escherichia–Shigella in the MA group and the P group were significantly decreased (*p* < 0.01 or *p* < 0.05), and the abundance of Bacteroidota was significantly increased (*p* < 0.01 or *p* < 0.05). The abundances of Proteobacteria and Escherichia–Shigella in the MAa group were significantly higher than those in the N, MA, and P groups (*p* < 0.01 or *p* < 0.05), and the abundances of Bacteroidota and Firmicutes in the MAa group were significantly lower than the other groups (*p* < 0.01 or *p* < 0.05).

### Effect of manual acupuncture on blood-brain barrier disruption

**FIGURE 3:**
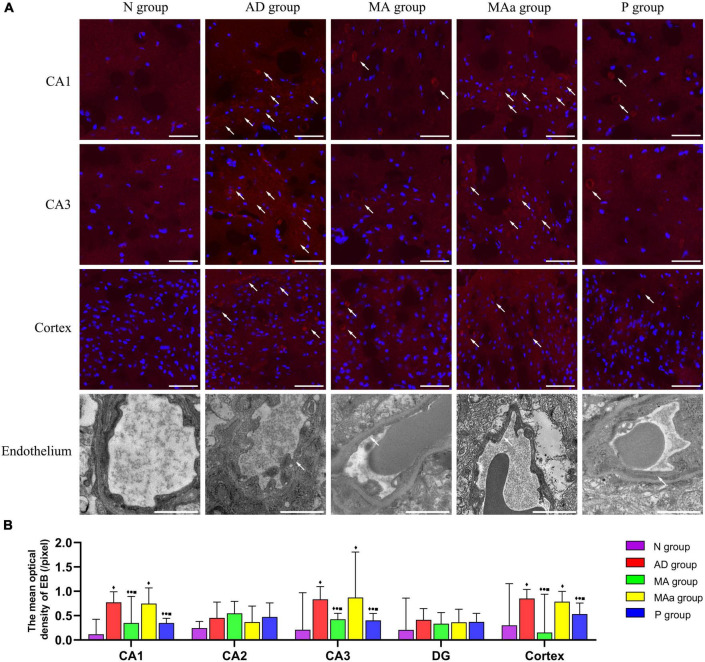
Results of EB extravasation and TEM analysis for each group (*n* = 6, mean ± SD). **(A)** Representative images of EB exudation and endothelial structure in each group (CA1, CA3, and cortex). Representative images of EB exudation in the CA2 and DG regions are shown in the Supplementary material. EB is labeled with red fluorescence. The nucleus is labeled with blue fluorescence. Scale bar is 20 μm. For endothelial structures, scale bar is 1 μm. **(B)** Comparison of the mean optical density in each group (CA1, CA2, CA3, DG, and cortex). Compared to the N group, ^◆^*p* < 0.01 or *p* < 0.05. Compared to the AD group, ^•^*p* < 0.01 or *p* < 0.05. Compared to the MAa group, ^■^*p* < 0.01 or *p* < 0.05.

The results of EB extravasation are shown in Figure 3. The EB dye was primarily radiolucent or clumped, and there was no obvious EB exudation in the hippocampi (CA1, CA2, CA3, and DG) or cortices of mice in the Nr group. However, different degrees of EB exudation were observed in the MA group, the MAa group and the P group. Significant and widespread EB exudation was observed in the CA1 and CA3 regions and cortex in the AD and MAa groups, and the mean optical intensity was significantly higher in the AD and MAa groups than the N group (*p* < 0.01). The mean optical intensity of EB in the CA1 and CA3 regions and cortex in the MA group and the P group was significantly lower than the AD group (*p* < 0.01) but it remained higher than the N group (*p* < 0.01). The mean optical intensity of EB in the CA1 and CA3 regions and cortex in the MA group and the P group was significantly lower than the MAa group (*p* < 0.05). No significant EB exudation was seen in the CA2 or DG regions, and fluorescent images of these two regions are shown in the Supplementary material. No significant differences in mean optical intensity were found in the CA2 and DG regions between the groups. Unlike the N group, the AD group and the MAa group exhibited a severely fractured capillary basement membrane with blurred edges and loss of tight junction structures. The MA group and the P group exhibited a clear and complete basement membrane with uniform density and improved tight junction structures.

### Effect of manual acupuncture on the expression of the blood-brain barrier-associated proteins

**FIGURE 4:**
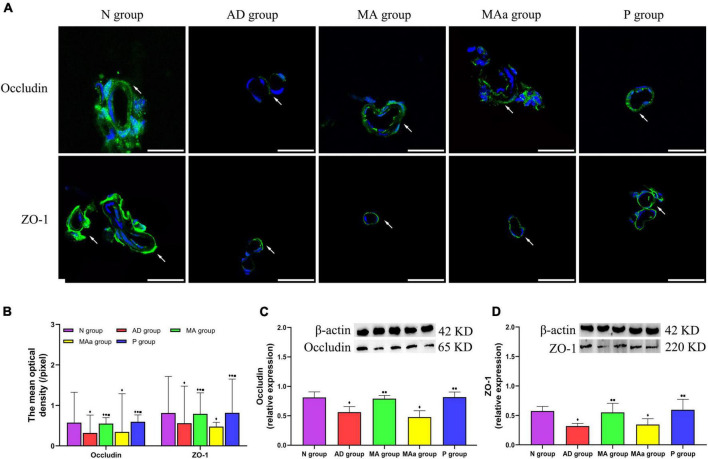
Results of the BBB-associated proteins in each group (*n* = 6, mean ± SD). **(A)** Representative images of IF staining of Occludin and ZO-1. Occludin and ZO-1 are labeled with green fluorescence. Scale bar is 50 μm. **(B)** Comparison of the mean optical density of Occludin and ZO-1 in each group**. (C,D)** Comparison of the relative expression of Occludin and ZO-1 in each group. Compared to the N group, ^◆^*p* < 0.01 or *p* < 0.05. Compared to the AD group, ^•^*p* < 0.01 or *p* < 0.05. Compared to the MAa group, ^■^*p* < 0.01 or *p* < 0.05.

The results of BBB-related proteins are shown in Figure 4. The tightly bound proteins ZO-1 and occludin were mainly distributed in the cerebrovascular endothelium in the Nr group and exhibited a ring-shaped distribution and tight intercellular connections. ZO-1 and occludin expression was more disorganized in the AD and MAa groups, a complete ring was not observed, and the staining intensity was decreased. However, the structure of ZO-1 and occludin was more complete in the MA group and the P group, and the fluorescence intensity was increased. The mean optical density and relative expression of occludin and ZO-1 in the AD and MAa groups were significantly lower than the N group (*p* < 0.01). The mean optical density and relative expression of ZO-1 and occludin in the MA and P groups were significantly higher than the AD and MAa groups (*p* < 0.01 or *p* < 0.05) but lower than the N group (*p* < 0.01). There were no differences in these measures between the AD and MAa groups.

### Effect of manual acupuncture on the levels of lipopolysaccharide and inflammatory factors

**FIGURE 5:**
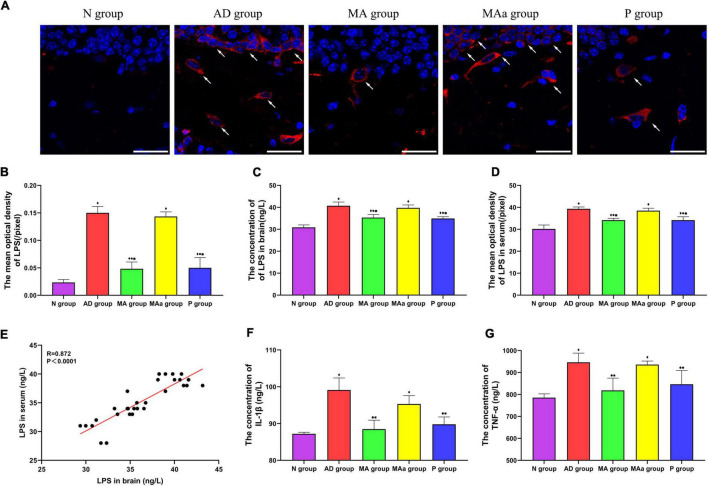
Results of LPS and inflammatory factor levels in each group (*n* = 6, mean ± SD). **(A)** Representative images of IF staining of LPS in each group. LPS is labeled with red fluorescence. The nucleus is labeled with blue fluorescence. Scale bar is 50 μm. **(B)** Comparison of the mean optical density of LPS in each group. **(C,D)** Comparison of LPS in serum and brain of all groups. **(E)** Pearson correlation analysis of LPS between brain and serum. **(F,G)** Comparison of serum TNF-α and IL-1β in all groups. Compared to the N group, ^◆^*p* < 0.01 or *p* < 0.05. Compared to the AD group, ^•^*p* < 0.01 or *p* < 0.05. Compared to the MAa group, ^■^*p* < 0.01 or *p* < 0.05.

The results of fluorescence staining of LPS in each group are shown in Figure 5. LPS was visible as an oval shape in the brain. No LPS was observed in the N group, but a large amount of LPS was present in the AD and MAa groups. The optical density in the AD and MAa groups was obviously higher than the Nr group (*p* < 0.01). The LPS content was decreased in the MA and P groups, and the optical density values were also significantly decreased compared to the AD and MAa groups (*p* < 0.01). The intracerebral LPS load and serum LPS load were higher in the AD and MAa groups than the N group (*p* < 0.01). The intracerebral and serum levels of LPS were significantly lower in the MA and P groups than the AD group and MAa group (*p* < 0.01). There was no significant difference between the AD and MAa groups. Pearson correlation analysis showed that a significant positive correlation between brain LPS and serum LPS levels (*r* = 0.872, *p* < 0.0001).

The concentrations of TNF-α and IL-1β in the serum were significantly higher in the AD and MAa groups than the Nr group (*p* < 0.01). The concentrations of these two inflammatory factors were significantly lower in the MA and P groups than the AD and MAa groups (*p* < 0.01 or *p* < 0.05).

## Discussion

The MWM test is an important tool for the evaluation of cognitive ability, and it is widely used to assess spatial learning and memory (Tian et al., 2019). The results of the MWM test indicated that the mice in the AD group exhibited a significant decrease in spatial learning and memory, which is consistent with previous studies (Ding et al., 2019a,b; Xu et al., 2020). Acupuncture and probiotics restored spatial cognition in APP/PS1 mice, and the effects of the two interventions were comparable. Previous work by our team found that acupuncture induced rapid and efficient improvement in cognitive ability in SAMP8 mice compared to donepezil (Ding et al., 2019b). However, acupuncture did not show better efficacy than probiotics in the current study. We hypothesized that this result was associated with different modulatory mechanisms of probiotics and donepezil. Unlike the modulation of acetylcholinesterase by donepezil, probiotics improve cognitive ability by promoting the production of neurotransmitters, such as GABA and brain-derived neurotrophic factor (BNDF), which is related to learning abilities (Athari Nik Azm et al., 2018; Kim et al., 2020; Goyal et al., 2021), alter the balance of pro-and anti-inflammatory cytokines to produce multitarget modulatory effects (Kesika et al., 2021). Previous studies found that acupuncture also exhibited multitargeting characteristics, such as anti-inflammation (Jiang et al., 2018; Cai et al., 2019), antagonism of amyloid Aβ (Wang et al., 2016; Zheng et al., 2021), modulation of oxidative stress (Liu et al., 2013; Chang et al., 2019), inhibition of apoptosis (Wang et al., 2009), and regulation of glucose metabolism (Huang et al., 2007; Cao et al., 2017; Xu et al., 2020). We believe the equal effects of acupuncture and probiotics on the modulation of cognitive ability may be associated with these multitarget characteristics. The current study reconfirmed that the intervention protocol is effectively improved cognitive ability, and the selection of acupoints and experimental protocol provide a reference for subsequent research.

The results of gut microbiota analysis showed that species diversity and composition were more altered in the AD group than the N group. This result suggested that gut microbiota dysbiosis occured in APP/PS1 mice by 6 months of age, which is consistent with the results of previous studies (Shen et al., 2017; Bäuerl et al., 2018). Compared to the AD group, the Sobs index and the abundance of the abovementioned phyla in the MA and P groups showed an opposite trend. This result showed that MA significantly regulated gut microbiota diversity and species composition in APP/PS1 mice, which was evidenced by a significant increase in the Sobs index and the abundance of Bacteroidota, and a decrease in the abundances of Firmicutes, Proteobacteria, and Escherichia–Shigella. Our subsequent studies will expand the sample and introduce macro-genomics to explore the specific connotation of acupuncture regulating gut microbiome disorders in AD and identify the targeted bacteria. Previous studies found that acupuncture decreased the abundance of *Bacteroides fragilis* and Streptococcus in Parkinson’s disease and knee osteoarthritis (Jang et al., 2020; Wang et al., 2021a). However, the regulatory effect of acupuncture on the abundances of different species observed in our study differed from that reported in other studies. We speculated that this difference may be due to specific gut microbiota changes in specific diseases. These results suggest that the regulatory effect of acupuncture on the gut microbiota is not limited to a certain phylum or genus, but may exhibit holistic and multitarget characteristics. Studies found that acupuncture enhanced 5-hydroxytriptamin (5-HT), which regulated gastrointestinal secretion and peristalsis (Zhan et al., 2014; Millan et al., 2020), and vasoactive intestinal peptide (VIP) which related to the gut sensitivity (Zhenzhong et al., 2015). Acupuncture also modulated intestinal microbial metabolites, such as glutamate and alanine metabolism (Yang et al., 2022). We speculated that the extensive non-specific regulatory effects of acupuncture on the gut microbiome were related to improvement of the local microenvironment, which deserves further investigation.

Blood-brain barrier dysfunction occurs in the early stage of AD (Zenaro et al., 2017). BBB dysfunction is characterized by a decrease in the expression of tight junction proteins (ZO-1 and occludin), leakage of fibrinogen from the brain parenchyma (Alvarez et al., 2011; Carrano et al., 2012), and the development of inflammation around the microvasculature (van de Haar et al., 2017; Yamazaki and Kanekiyo, 2017; Nandini et al., 2022). We observed significant EB exudation (CA1, CA3, and cortex), a significant reduction in the expression of tight junction proteins and basement membrane disruption in the AD group, which indicated that the BBB was significantly disrupted in APP/PS1 mice. EB exudation in the CA1 and CA3 regions and cortex was significantly reduced and tight junction structures were improved in the MA and P groups compared to the AD group. This finding indicated that MA significantly improved BBB disruption in APP/PS1 mice, and the effect of MA was comparable to probiotics. This study is the first report of the benign regulatory effect of acupuncture on structural and functional disruption of the BBB in AD, and results indicate that the BBB is an important target of acupuncture in the treatment of AD.

**FIGURE 6:**
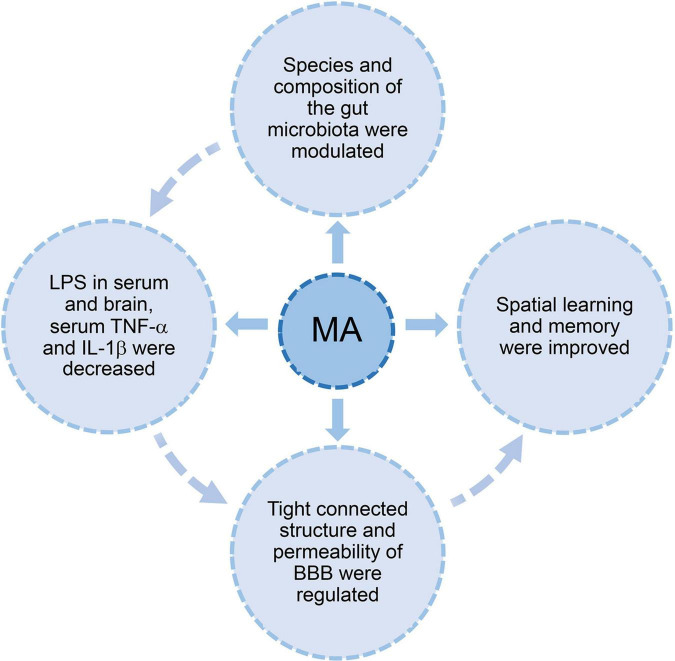
The hypothesis diagram about the relationship between acupuncture, gut microbiota, and BBB. Acupuncture modulated the diversity and species composition of gut microbiota. Intestinal LPS leakage diminished with the decrease in proinflammatory bacteria. This process may improve BBB dysfunction, which was evidenced by an increase in tight junction proteins and lower permeability. Cognitive ability improved with the recovery of BBB dysfunction. One possible mechanism of acupuncture regulation of BBB dysfunction *via* the gut microbiome is the reduction of LPS, TNF-α, and IL-1β.

Previous studies found that BBB dysfunction was closely related to gut microbiota dysbiosis, specifically an increase in the levels of Gram-negative bacteria and LPS (Zhan et al., 2016; Zhao and Lukiw, 2018; Marizzoni et al., 2020). LPS is an important pathological factor that leads to endothelial structure damage, and it is a potent proinflammatory mediator (Hauss-Wegrzyniak et al., 1998; Skelly et al., 2013; Asti and Gioglio, 2014; Mohammad and Thiemermann, 2020). LPS in the brains of AD patients is associated with Gram-negative bacteria (Zhan et al., 2018). LPS is released into the extracellular space when gut bacteria are destroyed (Brown, 2019). Serum LPS levels in AD patients were tripled compared to healthy individuals (Zhang et al., 2009). Our results showed that the levels of LPS and inflammatory factors (TNF-α and IL-1β) were significantly increased in the AD group. Pearson correlation analysis also demonstrated that LPS in serum and brain showed a positive correlation. Our results showed that MA reduced LPS and inflammatory factor levels, and the effect of MA was comparable to probiotics. Studies have shown that Gram-negative bacteria, especially Enterobacter–Shigella, are related to LPS (Cattaneo et al., 2017). Based on these gut microbiota result, we speculated that the modulation of LPS by acupuncture was related to the benign regulation of specific genera, such as Proteobacteria and Enterobacter–Shigella. previous study found that LPS containing *Escherichia coli (E. coli)* produced extracellular amyloid (Zhao et al., 2015a), and high levels of *E. coli* LPS were observed in hippocampal and temporal lobe neocortices from AD brains (Zhao et al., 2017). *E. coli* LPS was bound to brain endothelial cell receptors and produced inducible nitric oxide synthase (iNOS), which disrupted tight junctions to exacerbate BBB destruction (Al-Obaidi and Desa, 2018). In addition, *E. coli* LPS exerted a highly proinflammatory effect on neurons (Zhao et al., 2017). We inferred that the benign regulation of BBB dysfunction by acupuncture may be based on the modulation of LPS. Intestinal LPS may be a key molecule for acupuncture to regulate BBB disruption, which deserves further study. The relationship between acupuncture, gut microbiota, and BBB is shown in Figure 6.

To further explore the relationship between acupuncture-mediated regulation of the BBB and the gut microbiota, we used the MAa group as a negative control for the MA group. After antibiotic pretreatment, the gut microbiota of the MAa group was disrupted, and this change persisted until the end of the intervention. This dysbiosis primarily manifested as a decrease in species diversity and a significant increase in the abundances of Proteobacteria and Escherichia–Shigella. Antibiotics inhibited the benign regulatory effects of MA on cognitive ability, BBB disruption, LPS levels, and systemic inflammation. This result suggested that the gut microbiota may played an important role in the ability of acupuncture to improve cognition and alleviate BBB disruption, and the gut microbiota may be an important target for BBB modulation by acupuncture. Recent studies indicated that antibiotics, such as cephalosporin (Payne et al., 2017), streptozotocin (Ravelli et al., 2017), and ampicillin (Wang et al., 2015), increased the neuroinflammatory state and impaired cognitive function (Minter et al., 2016), which is consistent with the findings of this study. A number of studies also showed that some antibiotics were beneficial for cognitive function, by reducing neuroinflammation due to the dysbiosis of the gut microbiome. Rifampin and minocycline reduced inflammatory cytokines in the brain (Budni et al., 2016; Yulug et al., 2018). Rapamycin regulated cognitive deficits (Wang et al., 2014). We inferred that antibiotics were beneficial or detrimental in AD may depending on the type of antibiotic, animal strain, or the specific role of gut microbes. On the one hand, the specific effects of the antibiotic intervention on cognitive ability and BBB disruption in this study may be clarified by establishing an antibiotic group. Behavioral tests may also be added at the beginning and end of antibiotic gavage. On the other hand, sterile mice may also be introduced to more clearly show acupuncture adjustments of the BBB and gut microbiome.

Our study reconfirmed that MA improved the cognitive ability of APP/PS1 mice, and the effect of MA was similar to probiotics. We reported that MA had a benign regulatory effect on the gut microbiota in APP/PS1 mice. At the phylum level, Bacteroidota was upregulated, and Proteobacteria and Firmicutes were downregulated. At the genus level, Escherichia–Shigella was downregulated. We reported the benign regulatory effect of acupuncture on BBB disruption, LPS load and systemic inflammation. The decrease in LPS load and systemic inflammation play an important role in the regulation of BBB dysfunction. We speculated that the benign regulation of the BBB by acupuncture was may achieved *via* the gut microbiota. Escherichia–Shigella may be the target genus, but further studies are needed.

## Data availability statement

The datasets presented in this study can be found in online repositories. The names of the repository/repositories and accession number(s) can be found in the article/Supplementary material.

## Ethics statement

The animal study was reviewed and approved by the Animal Ethics Committee of Beijing University of Chinese Medicine.

## Author contributions

YZ and ND: experimental design, data analysis, and manuscript preparation. XH, YLZ, JZ, and YL: data collection. ZL: experimental design. All authors contributed to manuscript revision, read, and approved the submitted version.
