# Supplementary material for: Manual acupuncture benignly regulates blood-brain barrier disruption and reduces lipopolysaccharide loading and systemic inflammation, possibly by adjusting the gut microbiota
Source: Front Aging Neurosci. 2022 Oct 13;14:1018371. doi: 10.3389/fnagi.2022.1018371 (PMC9607933; doi:10.3389/fnagi.2022.1018371)
Supplement: Supplementary file 1 [file Data_Sheet_1.PDF]

Supplementary Material

1 Supplementary Figure

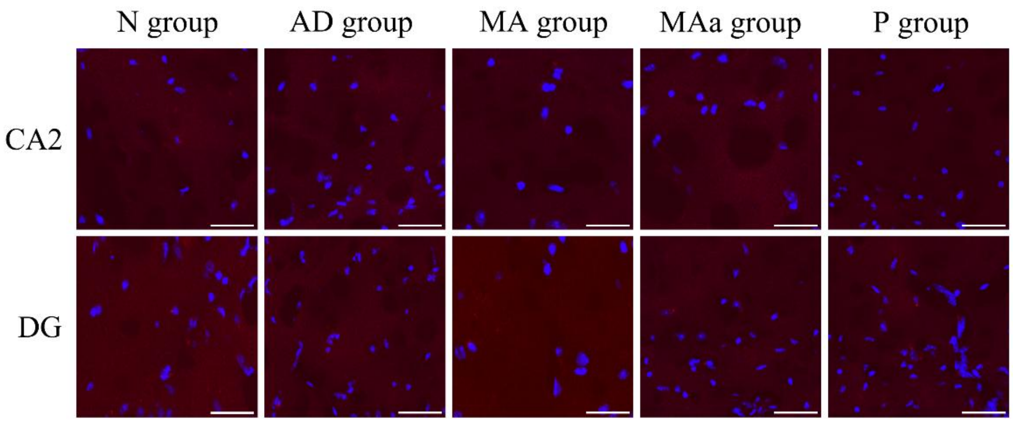

Supplementary Figure: Fluorescent images of EB exudation in the CA2 and DG regions of the hippocampus are shown in the figure. There is no obvious EB exudation in these two regions. For EB (red), scale bar is 20 µm.
